# High Frequency of Mayaro Virus IgM among Febrile Patients, Central Brazil

**DOI:** 10.3201/eid2306.160929

**Published:** 2017-06

**Authors:** Sandra Brunini, Divânia Dias Silva França, Juliana Brasiel Silva, Leandro Nascimento Silva, Flúvia Pereira Amorim Silva, Mariana Spadoni, Giovanni Rezza

**Affiliations:** Universidade Federal de Goiás, Goiânia, Brazil (S. Brunini);; Secretaria Municipal de Saúde, Goiânia (D.D.S. França, J.B. Silva, L.N. Silva, F.P.A. Silva);; Universidade Federal do Paraná, Paranà, Brazil (M. Spadoni);; Istituto Superiore di Sanità, Rome, Italy (G. Rezza)

**Keywords:** Alphavirus, Mayaro virus, chikungunya, Brazil, vector-borne infections, viruses, mosquitoes

## Abstract

Mayaro virus (MAYV), an *Aedes* mosquito–borne alphavirus, is endemic to Brazil and other South America countries. We investigated dengue- and chikungunya-negative febrile patients visiting rural areas near Goiânia, Goiás, and found a high proportion (55%) of MAYV IgM. Our findings suggest the presence of highly endemic foci of MAYV in central Brazil.

Mayaro virus (MAYV) is an *Aedes* mosquito–borne alphavirus of the New World, transmitted mainly by tree canopy–dwelling *Haemagogus* spp. mosquitoes, was discovered in Trinidad in 1954. MAYV causes a dengue-like acute febrile illness with arthralgic manifestations (*1*). Exposure to MAYV has been reported in several countries of Central and South America, and Mayaro fever has been identified in French Guiana, Suriname, Venezuela, Peru, Bolivia, and Brazil (*2*,*3*). In Brazil, Mayaro fever has been reported in the Amazon region (*2*,*4*) and in Mato Grosso (*5*,*6*).

At the end of 2014, after chikungunya virus (CHIKV) spread in South America, the Brazil Ministry of Health enhanced the surveillance system for dengue-like illness. Accordingly, febrile patients attending primary care centers are tested for dengue virus (DENV) infection by using viral antigen (NS1), virus isolation, or reverse transcription PCR (up to 5 days after symptom onset), and/or ELISA for IgM (after the sixth day). DENV-negative patients fitting the clinical and epidemiologic criteria for chikungunya set by the Ministry of Health (http://www.saude.gov.br) are notified to the Center for Strategic Information in Health Surveillance of Goiânia city for further investigation. 

Blood samples collected during June 1, 2014–June 30, 2015, were stored at −20°C and sent to the Department of Arbovirology and Hemorrhagic Fevers, Instituto Evandro Chagas (Belém, Brazil), to be evaluated for CHIKV by an IgM-capture ELISA (MAC ELISA; Centers for Disease Control and Prevention, Atlanta, GA, USA). A subsample of CHIKV-negative serum collected during December 2014–June 2015 was tested by hemagglutination inhibition (HI) (*7*) against the most common arboviruses. We evaluated serum samples with monotypical or heterotypic reactivity to alphaviruses, with HI titers >40 using an IgM MAC ELISA for MAYV, as described by Kuno (*8*) and then modified (*9*). The results were sent to the Center for Strategic Information in Health Surveillance for further clinical and epidemiologic investigations.

We tested 75 samples from DENV-negative patients for CHIKV. Five of the 31 DENV-negative persons from whom serum was collected during June–December 2014 and none of the 44 from January–June 2015 were positive for CHIKV IgM. Two additional samples yielded indeterminate results for CHIKV.

Of 27 CHIKV-negative samples tested by HI, 16 were reactive to alphaviruses (median titer 160 [range 80–1,280]); of these, 15 (56%) were confirmed as positive by IgM MAC ELISA for MAYV, and 1 sample was borderline. The median interval between symptom onset and serum collection was 37 days (range 12–167 days).

Patients were a median of 45 years of age (range 30–70 years). Fourteen were women. Patients’ educational level was high; 11 (73%) patients had >8 years’ education, and 47% had >12 years’ education.

All the patients with an antibody profile suggesting recent MAYV infection resided in Goiânia but had traveled to rural areas in the 15 days before symptom onset. Twelve patients had visited farms or small holdings in the forests around towns located 34 km (Hidrolandia) and 46 km (Bela Vista) from Goiânia (Technical Appendix Figure). In particular, 7 patients had been around Bela Vista and 5 in the area of Hidrolandia, whereas only 1 had been near Pontalina and 2 in Itacaja (Tocantins), which is outside the state of Goiás. Ten of these 11 patients reported engaging in recreational activities.

The high frequency of MAYV IgM detection among febrile patients in Goiânia is surprising. Identification of recent infections most likely acquired in rural areas and forests around the city of Goiânia indicates the existence of active foci where a sylvatic cycle of MAYV is established. The MAYV belt around Goiânia, where epizootics of jungle yellow fever were also reported (http://www.paho.org/hq/index.php?option=com_docman&task=doc_view&Itemid=270&gid=34247&lang=en), is represented by a wet area hosting small primates, which may play a role as virus amplifier.

Our conclusions are subject to several limitations. First, selection bias could affect findings regarding the proportion of MAYV recent infections because of the lack of systematic testing for dengue-like illness. Second, we did not conduct PCR to identify acute infections because the samples were collected after the viremic phase. Third, plaque-reduction neutralization testing was not performed, and because of the lack of convalescent serum, IgG seroconversion or titer increase were not evaluated; however, MAC ELISA is considered a valid technique for diagnosing recently acquired infection with MAYV (*10*). Finally, the frequency of rash (Table), higher than in other case series (*4*), might be overestimated because of stringent selection criteria used for MAYV testing.

**Table:** Clinical characteristics of 15 patients positive for IgM against Mayaro virus, Goiânia, Goiás, Brazil, June 2014–June 2015

Sign or symptom	No. (%) patients
Fever	15 (100)
Arthralgia	14 (93)
Joint edema	14 (93)
Skin Rash	14 (93)
Headache	13 (87)
Weakness	13 (87)
Myalgia	12 (80)
Eye pain	8 (53)
Icterus	4 (27)
Photophobia	3 (20)
Severe itching	3 (20)
Lymphadenopathy	2 (13)
Vomiting	2 (13)

In conclusion, infection with MAYV occurs more frequently than expected in central Brazil. Mayaro fever should be considered in the differential diagnosis with DENV, CHIKV, and Zika virus infections in areas characterized by arbovirus cocirculation.

Technical Appendix Goiânia, Goiás, Brazil, and the area where most cases of Mayaro virus infection were detected, June 1, 2014–June 30, 2015.
