# Supplementary material for: High Frequency of Mayaro Virus IgM among Febrile Patients, Central Brazil
Source: Emerg Infect Dis. 2017 Jun;23(6):1025–6. doi: 10.3201/eid2306.160929 (PMC5443426; doi:10.3201/eid2306.160929)
Supplement: Technical Appendix — Goiânia, Goiás, Brazil, and the area where most cases of Mayaro virus infection were detected, June 1, 2014–June 30, 2015. [file 16-0929-Techapp-s1.pdf]

Article DOI: https://dx.doi.org/10.3201/eid2306.160929

High Frequency of Mayaro Virus IgM among Febrile Patients, Central Brazil

Technical Appendix

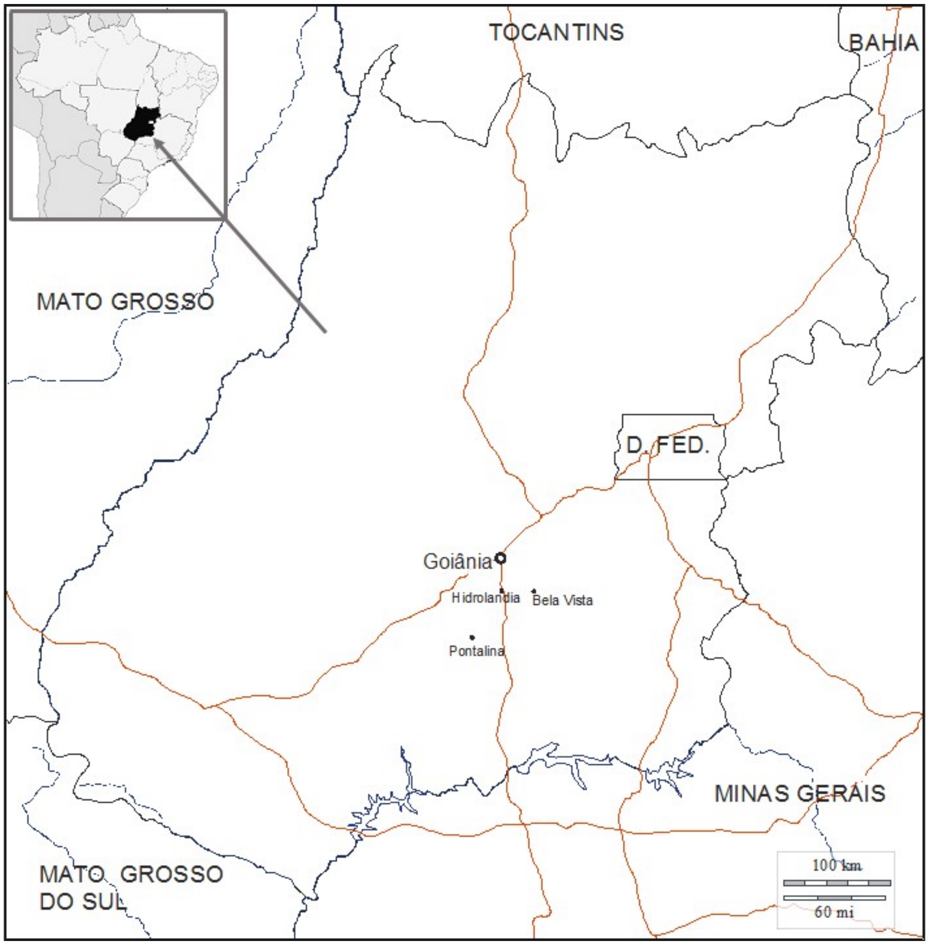

Technical Appendix Figure. Goiânia, Goiás, Brazil, and the area where most cases of Mayaro virus infection were detected, June 1, 2014–June 30, 2015.
